# Current understanding of the molecular mechanisms of chemotherapy-induced peripheral neuropathy

**DOI:** 10.3389/fnmol.2024.1345811

**Published:** 2024-04-10

**Authors:** Xinyu Chen, Yumeng Gan, Ngan Pan Bennett Au, Chi Him Eddie Ma

**Affiliations:** ^1^Department of Neuroscience, Hong Kong Special Administrative Region (HKSAR), City University of Hong Kong, Kowloon, Hong Kong SAR, China; ^2^School of Pharmacy and Biomedical Sciences, University of Portsmouth, Portsmouth, United Kingdom; ^3^Institute of Life Sciences and Healthcare, University of Portsmouth, Portsmouth, United Kingdom

**Keywords:** chemotherapy-induced peripheral neuropathy, dorsal root ganglion, mechanical allodynia, cold allodynia, intraepidermal nerve fibers

## Abstract

Chemotherapy-induced peripheral neuropathy (CIPN) is the most common off-target adverse effects caused by various chemotherapeutic agents, such as cisplatin, oxaliplatin, paclitaxel, vincristine and bortezomib. CIPN is characterized by a substantial loss of primary afferent sensory axonal fibers leading to sensory disturbances in patients. An estimated of 19–85% of patients developed CIPN during the course of chemotherapy. The lack of preventive measures and limited treatment options often require a dose reduction or even early termination of life-saving chemotherapy, impacting treatment efficacy and patient survival. In this Review, we summarized the current understanding on the pathogenesis of CIPN. One prominent change induced by chemotherapeutic agents involves the disruption of neuronal cytoskeletal architecture and axonal transport dynamics largely influenced by the interference of microtubule stability in peripheral neurons. Due to an ineffective blood-nerve barrier in our peripheral nervous system, exposure to some chemotherapeutic agents causes mitochondrial swelling in peripheral nerves, which lead to the opening of mitochondrial permeability transition pore and cytochrome c release resulting in degeneration of primary afferent sensory fibers. The exacerbated nociceptive signaling and pain transmission in CIPN patients is often linked the increased neuronal excitability largely due to the elevated expression of various ion channels in the dorsal root ganglion neurons. Another important contributing factor of CIPN is the neuroinflammation caused by an increased infiltration of immune cells and production of inflammatory cytokines. In the central nervous system, chemotherapeutic agents also induce neuronal hyperexcitability in the spinal dorsal horn and anterior cingulate cortex leading to the development of central sensitization that causes CIPN. Emerging evidence suggests that the change in the composition and diversity of gut microbiota (dysbiosis) could have direct impact on the development and progression of CIPN. Collectively, all these aspects contribute to the pathogenesis of CIPN. Recent advances in RNA-sequencing offer solid platform for *in silico* drug screening which enable the identification of novel therapeutic agents or repurpose existing drugs to alleviate CIPN, holding immense promises for enhancing the quality of life for cancer patients who undergo chemotherapy and improve their overall treatment outcomes.

## 1 Introduction

Cancer remains a leading cause of mortality around the world, with an estimated 19.3 million new cases and 10 million deaths from cancer in 2020 (Ferlay et al., 2021). Nevertheless, recent advances in cancer diagnosis and medical care result in a growing number of patients surviving cancer. In 2016, approximately 16.5 million cancer survivors were living in the United States with prevalence projected to approach 26.1 million by 2040 (Shapiro, 2018). Despite impressive clinical outcomes achieved with chemotherapy, patients who undergo chemotherapy continued to experience chronic pain throughout their lifetimes that largely affects their quality of life (Paice et al., 2016). Chemotherapy drugs target mainly the malignant cancer cells (mostly rapidly proliferating cells); however, some of these drugs also exhibit potent adverse effects on non-proliferating healthy cells, such as neurons (Colvin, 2019). In contrast to the central nervous system (CNS) which is well protected by a blood–brain barrier, the sensory neurons and axons in the peripheral nervous system (PNS) are more susceptible than the CNS to damage from chemotherapy drugs, due to the absence of an effective blood-nerve barrier in the PNS (Starobova and Vetter, 2017). The cumulative exposure to neurotoxic chemotherapy drugs frequently leads to a pathological condition which is commonly known as chemotherapy-induced peripheral neuropathy (CIPN) (Au et al., 2014; Chine et al., 2019a,b). Several chemotherapy agents are known to cause CIPN in cancer patients, including platinum-based compounds (e.g., cisplatin, carboplatin, and oxaliplatin), taxanes (e.g., paclitaxel, docetaxel, and cabacitaxel), vinca alkaloid (e.g., vinblastine and vincristine), epothilones (ixabepilone), bortezomib, and thalidomide (Brown et al., 2019; Zajączkowska et al., 2019). CIPN prevalence varies from 19 to 85%, and it is agent- and dose-dependent (Fallon, 2013; Colvin, 2019). In general, CIPN occurs acutely, and the symptoms may slowly improve after withdrawal of the chemotherapy agent. However, in severe CIPN cases, the symptoms of CIPN including numbness, paraesthesia, hypersensitivity to cold temperature, hyperalgesia in all four extremities, and muscle weakness can persist for months or even years after completion of chemotherapy which severely diminish the quality of life of patient (Flatters et al., 2017). CIPN is often progressive during and after treatment, resulting in dose reduction or even discontinuation of the life-saving chemotherapy regimens, which posed significant challenges to achieving high therapeutic efficacy and patient survival (Flatters et al., 2017). Currently, there is a lack of proven strategies or interventions to prevent the development of CIPN, and the evidence for effective drug treatment for established CIPN is very limited.

CIPN patients exhibit a range of predominantly sensory symptoms including paresthesia, numbness, and mechanical and/or cold allodynia. There are several clinical assessment scales which have been developed for objective and reliable evaluation of CIPN, such as common toxicity criteria of the national cancer institute (NCI-CTC), and the total neuropathy score (TNS) (Cavaletti et al., 2013). Electrophysiological studies including electromyography and nerve conduction velocity studies are also used for the assessment of patients with CIPN (Miltenburg and Boogerd, 2014). Typical sensory symptoms usually first appear in the four extremities within weeks or months following the chemotherapeutic drug administration (Starobova and Vetter, 2017). Paclitaxel and oxaliplatin are associated with acute symptoms generally develop within days after drug infusion (Loprinzi et al., 2011; Argyriou et al., 2013). CIPN patients frequently experience painful sensations such as spontaneous shooting, stabbing, or electric shock-like pain as well as mechanical or thermal hyperalgesia (Grisold et al., 2012). Motor and autonomic dysfunctions may also occur, but less frequently. Motor symptoms including muscle weakness, muscle wasting or distal muscle cramps, loss of tendon reflexes, gait and balance disturbances, and impaired motor movement often develop in severe cases (Zajączkowska et al., 2019). CIPN patients with motor disturbances usually find difficulty in wearing shirts, holding a pen, manipulating small objects, opening a bottle, walking stairs, and standing due to weakened muscles (Wang et al., 2019; Maihofner et al., 2021). On rare occasions, autonomic symptoms including blurred vision, hearing problems, memory loss, constipation, dizziness with positional changes, poor urinary functions, and reduced sexual performance, may also occur in CIPN patients (Mols et al., 2016; Wang et al., 2019). Patients treated with cisplatin or oxaliplatin develop progressively worsening of peripheral neuropathy in the months after the discontinuation of chemotherapy, a phenomenon known as coasting (Grisold et al., 2012). In some severe cases, CIPN patients display irreversible sensory and motor deficits even years after the withdrawal of chemotherapy. Therefore, the quality of life has significantly decreased and a better understanding of the underlying mechanisms of CIPN is needed.

Despite significant efforts to establish pre-clinical CIPN rodent models by using chemotherapy drugs which is known to induce CIPN in patients, there is still a lack of successful translation of research for treating CIPN. Nevertheless, pre-clinical animal models of CIPN not only greatly further our understanding of the mechanisms underlying CIPN neurotoxicity, but also establish basis for addressing important clinical questions and prioritizing clinical studies. Pre-clinical animal models also shed new light on possible mechanisms of CIPN, including abnormal cytoskeletal architecture, disrupted axonal transport, mitochondrial dysfunctions, altered neuronal excitability in peripheral neurons, and neuroinflammation. In this review, we summarize rodent models of CIPN, the applicability of animal behavioral tests, and our current understanding of pathogenic mechanisms underlying the development of CIPN.

## 2 Animal models for CIPN studies

Animal models are frequently used to study the pathogenesis of CIPN and more importantly, to evaluate the drug efficacy in preventing or reversing CIPN symptoms (Hoke and Ray, 2014). In some early studies, chemotherapeutic agents (paclitaxel) were locally injected into the sciatic nerves of rats (Roytta et al., 1984), leading to extensive demyelination, axonal swelling (Roytta and Raine, 1985), axonal loss, and muscle atrophy (Roytta and Raine, 1986). However, the route of administration of these studies is irrelevant to clinical settings as paclitaxel is usually administered systematically and never injected directly into the peripheral nerves of cancer patients. Therefore, subsequent research studies deliver chemotherapeutic drugs via intravenous or intraperitoneal injection, which is more clinically relevant.

In the past decades, pre-clinical animal models for CIPN studies are successfully established to study the neurotoxicity of chemotherapeutic agents, including paclitaxel (Polomano et al., 2001; Siau et al., 2006; Boehmerle et al., 2014; Chine et al., 2019a), vincristine (Siau et al., 2006; Boehmerle et al., 2014; Chine et al., 2019b), cisplatin (Joseph and Levine, 2009; Boehmerle et al., 2014), oxaliplatin (Joseph and Levine, 2009; Nassini et al., 2011), and bortezomib (Meregalli et al., 2010; Boehmerle et al., 2014). Mice and rats in different genetic background, age and gender are commonly used for CIPN studies. Despite little consistency in the type of animals being used for the CIPN study, most of the studies adopt a dosage regimen that simulates the chemotherapy treatment in clinical settings by repeated intraperitoneal or intravenous administrations of chemotherapeutic agents (Hoke and Ray, 2014). The commonly used animal species and dosage regimen of chemotherapy agents were summarized in detail (Table 1).

**Table 1:** Summary of treatment paradigm to induce chemotherapy-induced peripheral neuropathy (CIPN) in rodents.

Chemotherapeutic agents	Dosing paradigm	Route of administration	Species	References
Paclitaxel	1 mg/kg on 4 alternative days (days 0, 2, 4, and 6)	i.p.	C57BL/6 J mice ♂	Boehmerle et al. (2014) and Chine et al. (2019a)
	60 mg/kg on 3 alternative days (days 0, 2, and 4)	i.v. to jugular vein	C57BL/6 J mice ♀	Wang et al. (2004)
	2 mg/kg for 5 consecutive days	i.p.	CD1 mice ♂	Ruiz-Medina et al. (2013)
	2 mg/kg on 4 alternative days (days 0, 2, 4, and 6)	i.p.	Sprague–Dawley rats ♂	Polomano et al. (2001), Siau et al. (2006), and Xiao and Bennett (2012)
	8 or 16 mg/kg, one dose per week for 5 weeks	i.p.	Wistar rats ♀	Cavaletti et al. (2007)
	5 or 7 mg/kg for 5 consecutive days	i.v.	Sprague–Dawley rats ♀	Cliffer et al. (1998)
	15 or 18 mg/kg, one dose per week for 4 to 5 weeks			
	15 or 18 mg/kg on day 0 and 4			
Oxaliplatin	3 mg/kg, single dose	i.p.	C57BL/6 J mice ♂	Nassini et al. (2011) and Trevisan et al. (2013)
	3.5 mg/kg, twice per week for 4 weeks	i.v.	CD1, C57BL/6, DBA/2 J and Balb/c mice	Renn et al. (2011) and Marmiroli et al. (2017)
	4 mg/kg for 2 days	i.p.	Sprague–Dawley rats ♂	Kawashiri et al. (2012)
	2 mg/kg for 5 days	i.p.	Sprague–Dawley rats ♂	Xiao and Bennett (2012)
	2 mg/kg, single dose	i.v.	Sprague–Dawley rats ♂	Joseph and Levine (2009) and Nassini et al. (2011)
	2 mg/kg on day 0, 2, and 4	i.p.	Sprague–Dawley rats ♂	Miguel et al. (2019)
	5 mg/kg, single dose	i.v.	Wistar rats ♀	Alberti et al. (2020)
Cisplatin	2.3 mg/kg on days 0–4, and days 10–14 (i.e., break on days 5–9 post-injection)	i.p.	C57BL/6 J mice ♂	Boehmerle et al. (2014)
	10 mg/kg, once per week for 8 weeks	i.p.	CD1 mice ♂	Apfel et al. (1992)
	5 or 10 mg/kg, once per week for 7 or 8 weeks	i.p.	Swiss mice ♀	Verdú et al. (1999)
	2 mg/kg, twice per week for 4 weeks	i.p.	Wistar rats ♀	Carozzi et al. (2010)
	2 mg/kg, single dose	i.v.	Sprague–Dawley rats ♂	Joseph and Levine (2009)
Vincristine	0.05 mg/kg on days 0–4, 7–11 (i.e., break on days 5–6 post-injection)	i.p.	C57BL/6 J mice ♂	Chine et al. (2019b)
	0.2 mg/kg, single dose	i.p.	C57BL/6 J mice ♂	Boehmerle et al. (2014)
	2 mg/kg, twice per week for 8 weeks	i.p.	CD1 mice ♂	Apfel et al. (1993)
	0.02, 0.1 or 0.2 mg/kg on days 0-4, 7-11 (i.e., break on days 5-6 post-injection)	i.v.	Sprague–Dawley rats ♂	Aley et al. (1996)
	0.05 mg/kg for 10 consecutive days	i.p.	Sprague–Dawley rats ♂	Siau et al. (2006)
Bortezomib	0.4 mg/kg, 3 doses per week for 4 weeks	i.p.	C57BL/6 J mice ♂	Boehmerle et al. (2014)
	0.2, 0.5, or 1 mg/kg, single dose	i.p.	C57BL/6 J mice ♂	Trevisan et al. (2013)
	1 mg/kg, twice per week for 6 weeks	s.c.	Swiss OF1 mice ♀	Bruna et al. (2010)
	0.15 or 0.2 mg/kg, 3 doses per week for 8 weeks	i.v.	Wistar rats ♂	Meregalli et al. (2010)
	0.15 or 0.2 mg/kg, 3 doses per week for 4 weeks	i.v.	Wistar rats ♀	Cavaletti et al. (2007) and Carozzi et al. (2010)

i.p., intraperitoneal injection; i.v., intravenous injection; s.c., subcutaneous administration. BW, body weight; 2×/week, twice per week.

### 2.1 Assessment of mechanical allodynia

**Figure 1:**
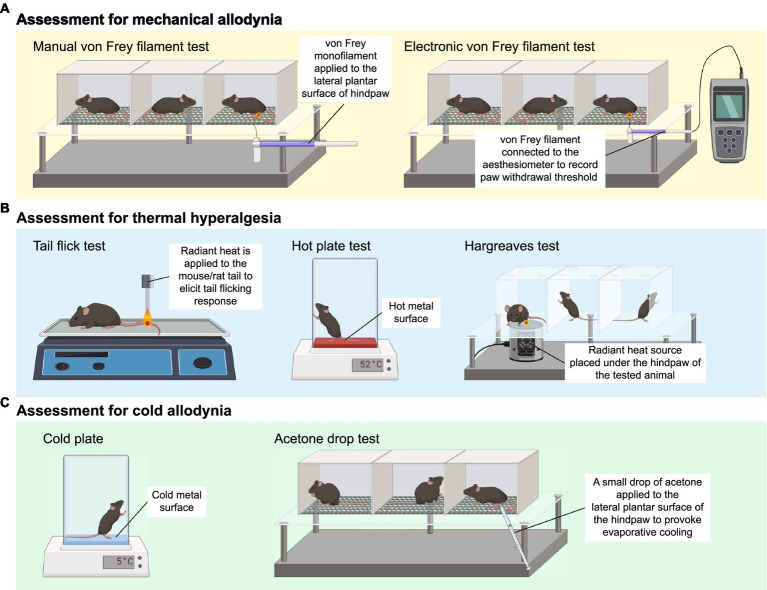
Animal behavioral assessment for the evaluation of peripheral neuropathy. **(A)** Both manual (left panel) and electronic von Frey filament tests are widely used to evaluate mechanical allodynia in mouse and rat models of chemotherapy-induced peripheral neuropathy (CIPN). Before the behavioral test, the tested animals are placed into wire mesh cages on an elevated platform for at least 30 min. For manual von Frey filament test (left panel), a set of von Frey monofilaments that are calibrated to deliver a fixed force is applied to the lateral plantar surface of the hindpaw for 2 s. A brisk paw withdrawal, licking or shaking is considered as a positive response to the stimulus. For electronic von Frey (right panel), the device delivers a gradually increasing force to the lateral plantar surface of the hindpaw. The force at which the animal shows a paw withdrawal response is recorded automatically by the aesthesiometer. **(B)** Tail flick test (left panel), hot plate test (middle panel) and Hargreaves test (right panel) are commonly used to assess thermal hyperalgesia. For tail flick test, a source of radiant heat is applied to the tail, and the time required to elicit tail flicking and twitch is recorded. For hot plate test, the tested animal is placed on a hot surface, and the latency for the animal to lick its hindpaw or jump out of the plate is recorded. For Hargreaves test, the tested animals are placed in an enclosed glass pane, and a radiant heat source is placed under the hindpaw of the animals. The latency for the animals to elicit a withdrawal response is recorded. **(C)** Cold plate test (left panel) and acetone drop tests (right panel) are widely used animal behavioral assessments for cold allodynia. For cold plate test, the tested animal is placed on a cold surface, and the time for the animal to provoke typical nociceptive responses (licking, paw withdrawal, shaking or jumping) is recorded. For acetone drop test, a small drop of acetone is applied directly to the hindpaw of the tested animal, and the duration of paw withdrawal, licking or flinching is recorded. BioRender.com

Mechanical allodynia is frequently observed in CIPN patients and defined as an exaggerated pain response to a normally innocuous stimulus (Ren, 1999). The assessment of mechanical allodynia is performed by using a set of von Frey monofilaments which provide a calibrated force to a portion of the subject’s body (Chaplan et al., 1994). For measurement of mechanical allodynia in mice or rats, animals are placed in a wire mesh-bottom cage and divided into individual compartments permitting free movement. A series of von Frey filaments are applied to the lateral plantar surface of the hindpaw for 2–5 s (Figure 1A). A brisk paw withdrawal, licking or shaking is considered as a positive response to the stimulus. Several commonly used methods to determine mechanical nociceptive threshold by measuring the paw withdrawal threshold (PWT), including “up-and-down” method (Dixon, 1965; Chine et al., 2019a), “ascending stimuli” method (Scholz et al., 2005) and “percent response” method (Chaplan et al., 1994). For “up-and-down” method, it starts at a filament estimated to be close to 50% withdrawal threshold. The next filament with higher force is applied if no positive response is elicited; or the next filament with lower force is applied if a positive response is obtained. The pattern of responses is recorded and 50% PWT is calculated, indicating that 50% of mice are responded to this 50% withdrawal threshold (Dixon, 1965; Chine et al., 2019a). A maximum of 6–8 stimuli are applied to obtain 50% PWT to avoid oversensitization of the animals. For “ascending stimuli” method, the lateral plantar surface of hindpaw is stimulated with filaments with ascending force starting from the filament with the lowest force (repeated 5 times for confirmation). The withdrawal threshold is then determined as the lowest force that is required to provoke a brisk paw withdrawal with at least 40–60% of response rate (Scholz et al., 2005). For “percent response” method, von Frey monofilaments with ascending force are applied to lateral plantar surface of hindpaw. For each filament, the number of applications remains constant (5–10 applications in general), and the number of positive responses (out of those 5–10 applications) is converted into “percent response.” The median 50% PWT is then calculated (Chaplan et al., 1994).

Electronic von Frey device operates in a similar way to manual von Frey filament test. Instead of a series of von Frey monofilaments with distinct force, electronic von Frey delivers a gradually increasing force with a single filament (Figure 1A). The force at which the animals exhibit the paw withdrawal response is automatically recorded by an aesthesiometer (Chine et al., 2019b). The total number of filament applications is dramatically reduced in electronic von Frey when compared with manual von Frey filament test. This is advantageous as electronic von Frey prevents the animals from receiving excessive pinches and become sensitized to a pinch (Deuis et al., 2017). Reduction in PWT measured by both electronic and manual von Frey filament tests serve as an indicator of mechanical allodynia (Chine et al., 2019a,b).

### 2.2 Assessment of thermal hyperalgesia

Three major behavioral assessments are widely used by researchers to assess thermal hyperalgesia in rodents, including tail flick test, hot plate test and Hargreaves test (Figure 1B). Tail flick test involves the application of heat stimuli to the tail of a loosely restrained rodent in the form of radiant heat or immersing the tail into a series of water baths at 46–52°C (D’Amour and Smith, 1941). The time required to elicit tail flicking and twitching is recorded. The hot plate test determines thermal hyperalgesia by measuring the nociceptive latency to lick a hindpaw or jump out of the enclosure when the mice or rats are placed on a metal surface with a constant temperature (50–55°C). To prevent prolonged exposure to noxious stimuli and minimize the risk of tissue damage to the hindpaw, a cut-off time of 20–40 s is commonly used (Deuis et al., 2017; Al-Romaiyan et al., 2023; da Motta et al., 2023; Li et al., 2023). Alternatively, the nociceptive threshold can be measured by counting the total number of flinches over a period of time at a given temperature (Deuis et al., 2013). For Hargreaves test, the animals are placed in an enclosed glass pane, thermal heat stimulus is delivered from a radiant or infrared source at a fixed distance to the plantar surface of the hindpaw. A hindpaw withdrawal at the site the heat stimulus is directed at, which is considered a reaction to the thermal stimulus. Hargreaves heat threshold and thermal latency to elicit a withdrawal response are recorded (Cheah et al., 2017). A rodent with thermal hyperalgesia showed a marked reduction in the latency to provoke a withdrawal response and increase flinching behavior over a set period of time (Minett et al., 2011; Deuis et al., 2013).

### 2.3 Assessment of cold allodynia

The cold plate test is one of the simplest methods to assess cold-evoked behavioral responses in mice and rats. Similar to the hot plate test, animals are placed on the pre-cooled plate at a specific temperature (usually 4–10°C) for a maximal observation period of 20–30 s to minimize the risk of tissue damage (Marcotti et al., 2023; Sun et al., 2023). The time for the animals to provoke typical nociceptive responses (licking, paw withdrawal, shaking or jumping) is then recorded (Figure 1C). Alternatively, the number of paw flinches over a set period of time is manually counted at a specific temperature (Ta et al., 2009, 2010; Deuis et al., 2013). In general, healthy mice do not elicit any typical nociceptive responses at an innocuous temperature of 10°C, but only respond to noxious cold stimuli when the temperature goes down to 4–5°C (Allchorne et al., 2005; Descoeur et al., 2011). However, mice treated with CIPN-inducing agents such as oxaliplatin and paclitaxel provoke nociceptive behaviors even at a relatively higher temperature (10°C) (Deuis et al., 2013; Marcotti et al., 2023; Sun et al., 2023), suggesting the presence of cold hypersensitivity as a result of CIPN.

Another widely used behavioral assessment to examine cold allodynia in rodents is the acetone drop test (Figure 1C). A small drop of acetone is applied directly to the hindpaw of the tested animals, which induces evaporative cooling on the hindpaw skin to an innocuous temperature between 15 and 21°C (Deuis et al., 2017). The duration of paw withdrawal, licking or flinching is manually recorded (Chine et al., 2019a,b; Warncke et al., 2021). Development of cold allodynia exhibits significantly increased paw flinching and latency as measured by the cold plate test and acetone drop test.

### 2.4 Electrophysiological assessment of CIPN

Behavioral assessment for mechanical allodynia, thermal hyperalgesia and cold allodynia can be subjective in which the researchers often require intensive training and proper blinding during the data collection. Nonetheless, electrophysiological assessment offers an objective evaluation of CIPN and treatment efficacy (Cliffer et al., 1998). In general, stimulating and recording surface electrodes are placed on the targeted nerves of CIPN patients, and SNAP amplitudes and NCV are recorded after stimulation to the targeted nerves (Tavee, 2019; O’Bryan and Kincaid, 2021). CIPN patients usually display a significant decrease in sensory nerve action potential (SNAP) and nerve conduction velocity (NCV) (Argyriou et al., 2005; McHugh et al., 2012; Matsuoka et al., 2016), suggesting the presence of peripheral neuropathy. For example, instances of moderate and severe CIPN symptoms were observed in cancer patients following treatment with platinum-based chemotherapy, paclitaxel, vincristine, or bortezomib. The severity of CIPN symptoms demonstrated a strong correlation with the extent of reduction in SNAP measured from the sural nerves (Matsuoka et al., 2016). Similarly, significant reductions in SNAP amplitudes and NCV were observed in the median nerves, ulnar nerves and sural nerves of patients treated with CIPN-causing agents (Myftiu et al., 2022). Consistent with clinical observations, we and others detected a marked reduction in SNAP amplitudes and NCV from the caudal tail nerve in paclitaxel-treated or vincristine-treated mice (Chine et al., 2019a,b; Bosanac et al., 2021). Therefore, a decrease in SNAP and NCV suggests a progressive axon degeneration and substantial demyelination under the pathological condition of CIPN (Chung et al., 2014).

Conventional SNAP and NCV measurement offer insights into the pathophysiology of the measured nerves – to identify whether it involves axon loss or demyelination, as indicated by distinct electrodiagnostic patterns (Tavee, 2019; O’Bryan and Kincaid, 2021). However, these conventional methods fall short in discriminating the specific source of axon loss, such as distinguishing between unmyelinated C-fibers and myelinated Aδ-fibers. To address this limitation, cutaneous nociceptive fibers are selectively stimulated by delivering electric stimuli to the superficial layer of the dermis. This approach enables a quantitative assessment of pain-related evoked action potential and NCV specifically from nociceptive Aδ-fibers, without stimulating other non-nociceptive fibers (Obermann et al., 2008).

### 2.5 Histological assessment of IENF density

The sensory disturbance observed in CIPN patients is often associated with the loss of sensory nerve fibers, as reflected by a prominent reduction in intraepidermal nerve fibers (IENFs) in skin biopsies and mouse model of CIPN (Siau et al., 2006; Boyette-Davis et al., 2011; Bechakra et al., 2018; Anand et al., 2019; Chine et al., 2019a,b). The IENFs consist of unmyelinated C-fibers and to a lesser extent thinly myelinated Aδ axonal fibers, which innervate the skin and are responsible for conveying touch, pain and thermal sensations. In the case of CIPN, the distal ending of IENFs undergo Wallerian degeneration and develop aberrant electrical activity, known as ectopic firing (Devor, 2009; Kocot-Kepska et al., 2021). Ectopic firing is defined as the generation of abnormal electrical signals by the damaged IENFs, which has significant implications for development and maintenance of a phenomenon called central sensitization. This amplification of pain signaling within the CNS involves an increased responsiveness of sensory neurons in the spinal cord and higher brain centers to sensory inputs (Woolf, 1983; Latremoliere and Woolf, 2009; Oaklander and Fields, 2009). Ectopic firing of central neurons triggers and sustains central sensitization, leading to the amplification and perpetuation of pain signals, and the persistence of chronic pain. In mice, the IENF density can be determined by counting the number of protein gene product 9.5 (PGP9.5)-positive sensory axonal fibers crossing the dermal-epidermal junction and then normalized with the length of epidermal layer (Chine et al., 2019a,b). Neuroprotective agents such as minocycline and overexpression of heat shock protein 27 showed promising beneficial effects to protect the IENFs from axonal degeneration and reverse mechanical and cold allodynia after treated with paclitaxel or vincristine (Boyette-Davis et al., 2011; Chine et al., 2019a,b).

### 2.6 Potential shortcomings in the validity of pain behavioral assessments and animal models of CIPN

These are commonly used and well-established animal behavioral assessments to evaluate “pain-like” behaviors in rodents, which closely resemble the clinical symptoms of CIPN observed in patients. There are a number of factors affecting the comparability of animal behavioral results between laboratories and even within the same laboratories, such as human bias, animal husbandry (i.e., housing isolation or overcrowding and enrichment), environmental stress (i.e., testing room architecture), and habituation (Crabbe et al., 1999; Pham et al., 2010; Cornelio et al., 2011; Langford et al., 2011; Bohlen et al., 2014). For instance, determination of a brisk withdrawal response due to the stimuli itself but not grooming behavior which requires intensive training (Hoke and Ray, 2014; Deuis et al., 2017). Habituation to the testing room before each session of animal behavioral assessments is necessary to minimize environmental stress. Given its inherently subjective nature of measuring pain in rodents, the researchers should be blinded to the treatment groups for the entire course of animal behavioral assessments. It is suggested that researchers should use more than one behavioral assessment to evaluate stimulus-evoked pain behaviors to validate reproducibility of findings and efficacy of drug treatments in animal models of CIPN. In recent years, video-based automated pain recognition with the aid of machine learning has been developed to objectively assess pain in rodents (Fried et al., 2020). Imaged the behavior of freely moving mice in an enclosed box and their home cage, to identify non-stimulus-evoked pain-related behaviors such as paw biting, licking and facial grimacing (Roughan et al., 2009; Brodkin et al., 2014; Burand et al., 2023). Luminance-based paw surface contact detection allows the measurement of force applied to a smooth surface and detection of the avoidance of contact due to pain (Zhang Z. et al., 2022). The use of automated behavioral analyses has been largely explored in acute pain; however, the robustness in determining chronic pain such as neuropathic pain remains elusive.

It should be noted that most of the CIPN studies have been conducted on healthy rodents without cancer, where a chemotherapeutic agent is administered to otherwise healthy animals. This lack of cancer presence in the animals cannot be directly comparable to cancer patients who receive chemotherapy for cancer treatment. In rodent, intraperitoneal administration of chemotherapy drugs is often used; however, it is rarely used in human patients except for the hyperthermic intraperitoneal chemotherapy procedure. This disparity in administration routes further amplifies the translation gap between preclinical and clinical efficacy. Furthermore, the use of animal models of CIPN in preclinical studies relies on oversimplified experimental designs that may not reflect the clinical reality of CIPN. Factors such as metabolic rates, dosing regimens, and treatment duration are significantly different between animal models and human patients, which further contributing to the translational gap.

## 3 Molecular mechanisms underlying the development of CIPN

### 3.1 Disruption of the neuronal cytoskeletal architecture and axonal transport

**Figure 2:**
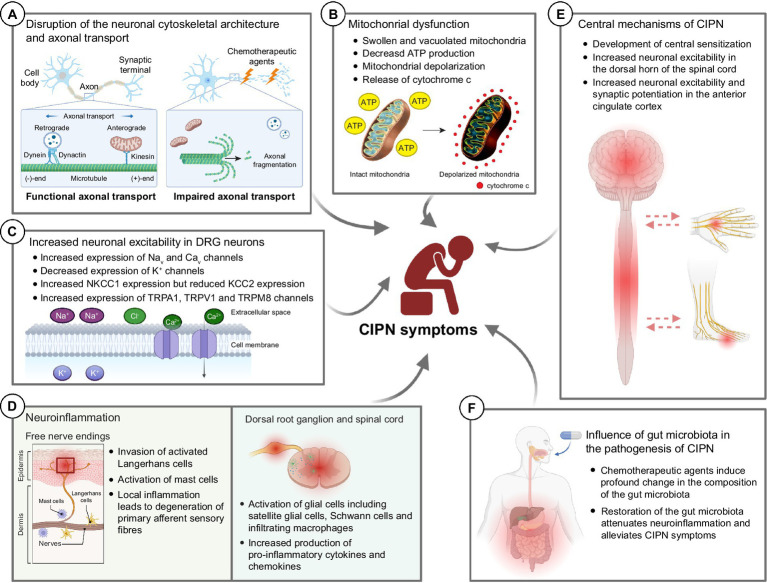
Potential molecular mechanisms underlying the development of chemotherapy-induced peripheral neuropathy (CIPN). **(A)** Chemotherapeutic agents induce drastic changes in the neuronal cytoskeletal architecture, resulting in axonal fragmentation in the sensory neurons. The disrupted microtubule cytoskeleton and integrity of sensory neurons also impaired the axonal transport of mitochondria and mRNA. **(B)** Chemotherapeutic agents induce mitochondrial swelling and vacuolation in both myelinated and unmyelinated sensory fibers in the peripheral nerve, resulting in reduced ATP production in the mitochondria. Also, chemotherapeutic agents induce rapid mitochondrial depolarization and cytochrome c release from mitochondria. **(C)** Chemotherapeutic agents induce significant changes in the expression of various ion channels—increased expression of Na_v_, Ca_v_, NKCC1 and TRP channels while decreased expression of K^+^ channels. These collectively increase the neuronal excitability in DRG neurons leading to pain hypersensitivity. **(D)** Chemotherapeutic agents induce local inflammation in the epidermis of glabrous hindpaw skin, resulting in the degeneration of primary afferent sensory fibers. Also, chemotherapeutic agents induce neuroinflammation in the dorsal root ganglion and dorsal horns of the spinal cord, leading to augmented production of pro-inflammatory cytokines and chemokines. **(E)** It is believed that chemotherapeutic agents also lead to the development of central sensitization, an increase in the responsiveness to nociception in the CNS to afferent inputs, which in turn leads to increased neuronal excitability in the spinal dorsal horn and brain (e.g., anterior cingulate cortex). **(F)** Recent findings proposed that chemotherapeutic agents induce a drastic change in the gut microbiota which eventually led to the development of pain hypersensitivity in CIPN patients. Therapeutic interventions such as probiotics aiming to restore the gut microbiota composition alleviated the mechanical and cold hypersensitivity after treatment of CIPN-causing agents. BioRender.com

Chemotherapeutic agents such as paclitaxel and vincristine are known to possess their antineoplastic effects by promoting (paclitaxel) or inhibiting (vincristine) microtubule assembly (Himes et al., 1976; Black, 1987), leading to cell cycle arrest and eventually apoptosis of malignant cells (Jordan, 2002). However, these antineoplastic agents are able to cross the blood-nerve barrier and bind to the β-tubulin (the building block of cytoskeleton in neuronal cells) of the peripheral sensory neurons and sensory nerve fibers (Windebank and Grisold, 2008). It adversely affects the cytoskeletal architecture of the healthy peripheral neurons such as dorsal root ganglions (DRGs) in cancer patients (Figure 2A). Our previous study demonstrated that both paclitaxel and vincristine drastically altered the cell mechanical properties of cultured DRG neurons due to excessive tubulin polymerization (paclitaxel) or depolymerization (vincristine). Subsequent high-resolution confocal microscopy confirmed that vincristine induced cell cytoskeleton disorganization in which the DRG cell bodies became more porous, and microtubules were loosely packed after vincristine treatment. This is consistent with the increased cell surface roughness of DRG neurons as detected by atomic force microscopy. As a result, the DRG neurons failed to extend their neurite when cultured with vincristine (Au et al., 2014). Similarly, treating the DRG neurons with paclitaxel significantly hampered microtubule dynamics, promoted the formation of retraction bulb-like structures at the distal growing tips (Gornstein and Schwarz, 2017), and thus markedly impaired the neurite outgrowth of cultured DRG neurons (Chine et al., 2019a). Interestingly, bortezomib, a potent proteasome inhibitor known to increase microtubule polymerization in neuronal cell line (Poruchynsky et al., 2008), also induced excessive microtubule polymerization in the cell bodies of DRG neurons by increasing hyper-stable delta 2 tubulin (D2) (Pero et al., 2021). Bortezomib treatment largely reduced the extent of neurite outgrowth and induced substantial axonal fragmentation in cultured DRG neurons (Staff et al., 2013). Similarly, accumulation of hyper-stable D2 by gene silencing of tubulin tyrosine ligase (TTL), an enzyme that re-tyrosinates tubulin to enhance microtubule dynamics, resulted in axonal fragmentation in DRG neurons (Pero et al., 2021). There is sufficient evidence that chemotherapeutic agents such as paclitaxel, vincristine and bortezomib caused axonal degeneration of IENFs (Bennett et al., 2011; Chine et al., 2019a,b; Geisler et al., 2019). The loss of the plasticity in remodeling axonal terminals might trigger the degeneration of distal sensory nerve fibers (i.e., IENFs) (Gornstein and Schwarz, 2017), resulting in altered pain perceptions and hypersensitivity (Thomas et al., 2023). It is important to note that these changes in neuronal cytoskeletal architecture were mostly observed in *in vitro* cultures of primary sensory neurons. In the actual *in vivo* setting, neurotoxic effects of CIPN-causing agents are likely to induce cellular changes not only in neurons, but also in other cell types that contribute to the development of CIPN.

Microtubule cytoskeleton and integrity are crucial for proper axonal transport of mitochondria, lysosome, mRNA, and axonal cargoes (Millecamps and Julien, 2013). Disruption of axonal transport in the peripheral neurons induces axonal degeneration in CIPN (Tourtellotte, 2016). Growing evidence suggests that chemotherapeutic agents markedly impaired the axonal transport (Bobylev et al., 2015; Berbusse et al., 2016; Van Helleputte et al., 2018). While axonal transport of mitochondria and lysosomes remained largely unaffected in cultured DRG neurons after paclitaxel treatment (Bobylev et al., 2015; Gornstein and Schwarz, 2017); however, the axonal transport of mRNA is significantly impaired (Bobylev et al., 2015). In a separate study, bortezomib impaired the axonal transport of mitochondrial in cultured DRG neurons (Staff et al., 2013), possibly via the accumulation of hyper-stable D2 in the neurons (Pero et al., 2021). Similarly, treating the cultured DRG neurons with 0.1 nM or 0.4 μM of vincristine (Berbusse et al., 2016; Van Helleputte et al., 2018), a dose that markedly reduced neurite outgrowth (Chine et al., 2019b), stalled the axonal transport of mitochondria that led to neurite degeneration (Berbusse et al., 2016; Van Helleputte et al., 2018). Treating the cultured DRG neurons with ACY-738 or tubastatin A, a small molecule that specifically inhibits HDAC6 (Butler et al., 2010; Jochems et al., 2014), successfully restores mitochondrial transport deficit induced by vincristine. More importantly, administration of ACY-738 or tubastatin A protected the mice against vincristine-induced loss of IENFs and mechanical allodynia (Van Helleputte et al., 2018). The results of these studies point to the direction that by maintaining the microtubule dynamics and promoting axonal transport in sensory neurons could protect the patients from axon degeneration and development of CIPN. Our previous study demonstrated that mitochondrial fusion promoter M1 promoted mitochondrial trafficking in the DRG neurons as well as in sciatic nerves leading to a remarkable axon regeneration (an energy-demanding process that requires robust mitochondrial transport along the regenerating axons) after peripheral nerve and optic nerve injuries (Au et al., 2022a).

### 3.2 Mitochondrial dysfunction

Mitochondria are the powerhouse of a cell and play a key role in the production of cellular ATP to support all forms of work in the body. Mitochondria also play an important role in maintaining intracellular calcium levels under normal physiological conditions (Kann and Kovacs, 2007; Misgeld and Schwarz, 2017). Neurons are vulnerable and susceptible to mitochondrial dysfunction, especially they have exceptionally high demand for energy to support the proper functions of neurons, including maintenance of resting potential, electric signal transduction and propagation, and neurotransmission on both pre- and post-synaptic vesicles (Misgeld and Schwarz, 2017). Due to the polarized cytoarchitecture of a neuron, maintaining a constant and hemostatic energy supply to peripheral neurons with exceedingly long axons (up to 1 m long) remained a highly challenging task involving effective anterograde and retrograde axonal transport of mitochondria (Saxton and Hollenbeck, 2012; Mandal and Drerup, 2019; Cheng et al., 2022). Failure to maintain efficient mitochondria ATP/ADP exchange and axonal transport of mitochondria often results in an energy crisis and axonal degeneration in energy-demanding neurons (Misgeld and Schwarz, 2017; Cheng et al., 2022).

Over the past two decades, accumulating evidence suggests that mitochondrial dysfunctions and impaired axonal transport of mitochondria are linked to the pathogenesis of CIPN (Trecarichi and Flatters, 2019; Doyle and Salvemini, 2021; Figure 2B). Platinum-based compounds including cisplatin and oxaliplatin, directly binds to the DNA and form platinum adducts in proliferating malignant cells, which in turn inhibits DNA replication that kill the cancer cells (Dasari and Tchounwou, 2014). However, cisplatin exhibited its off-target effects in peripheral neurons by direct binding to the mitochondrial DNA with the same affinity to the nuclear DNA in the DRG neurons, which interfered with replication and transcription of the mitochondrial DNA (Podratz et al., 2011). Due to the lack of DNA repair machinery within the mitochondria, cisplatin-induced mitochondrial DNA damage could not be repaired, which led to the release of cytochrome c from damaged mitochondria and induced apoptosis in DRG neurons (Gill and Windebank, 1998; McDonald and Windebank, 2002). In a rat model of bortezomib-induced peripheral neuropathy, mitochondrial swelling and vacuolization were observed in the primary afferent myelinated A-fibers and unmyelinated C-fibers. Swollen and vacuolated mitochondria in the bortezomib-treated sciatic nerve showed a markedly reduced capacity in ATP production, demonstrating a potent mitotoxic effect of bortezomib in the peripheral nerve. Similarly, paclitaxel binds with β-tubulin, the main component of the mitochondrial membrane, and opens the mitochondrial permeability transition pore (mPTP), resulting in a rapid mitochondrial depolarization and cytochrome c release from mitochondria in a neuroblastoma cell line (Andre et al., 2000; Carre et al., 2002). In line with the *in vitro* studies, paclitaxel-induced mitochondrial dysfunction significantly hampered cellular respiratory function and ATP production in DRG neurons (Duggett et al., 2017), leading to neuronal apoptosis (Chine et al., 2019a). In the distal sciatic nerve, paclitaxel induced mitochondrial swelling in both myelinated and unmyelinated axonal fibers and triggered a massive demyelination (Flatters and Bennett, 2006; Chine et al., 2019a). Another microtubule-binding agent vincristine also triggered a rapid mitochondrial depolarization in the distal axonal tips of cultured neurons (Ikegami and Koike, 2003; Chine et al., 2019b). Similar to paclitaxel, vincristine also induced profound mitochondrial swelling and widespread demyelination in the distal nerve of mice (Chine et al., 2019b). Interestingly, overexpression of human (h) Hsp27, a chaperone protein known to promote axon regeneration and function recovery after peripheral nerve injury (Ma et al., 2011; Asthana et al., 2021), not only protected the cultured DRG neurons from mitochondrial dysfunctions after vincristine treatment (Chine et al., 2019b), but also prevented the mitochondrial swelling in both myelinated and unmyelinated axonal fibers of sciatic nerves, and apoptosis in the DRG neurons (Chine et al., 2019a,b). More importantly, by restoring mitochondrial integrity via overexpressing hHsp27, the development of mechanical and cold allodynia was completely prevented in paclitaxel-treated or vincristine-treated hHsp27 overexpressing mice (Chine et al., 2019a,b). Administration of acetyl-l-carnitine, a compound that is shown to effectively reduce oxidative stress and improve mitochondrial function in aging rats (Liu et al., 2002), protected the peripheral neurons from mitochondrial dysfunctions and paclitaxel−/oxaliplatin−/bortezomib-induced peripheral neuropathy (Zheng et al., 2011; Xiao and Bennett, 2012; Xiao et al., 2012; Zheng et al., 2012). Collectively, these studies demonstrate that by targeting mitochondrial integrity and normal mitochondrial function in peripheral neurons, it represents a new therapeutic strategy for treating CIPN.

### 3.3 Increased neuronal excitability in DRG neurons

DRG neurons express a variety of ion channels including, voltage-gated sodium channels (Na_v_), voltage-gated potassium channels (K_v_), voltage-gated calcium channels (Ca_v_), chloride channels and transient receptor potential (TRP) channels. All these ion channels are associated with pain-sensing and control of the inherent excitability (Costigan et al., 2009; Stevens and Stephens, 2018). Free nerve endings at musculoskeletal afferents are responsible for the detection of somatic pain. Upon noxious stimuli, pain signals are transduced at the nerve terminals through a specialized set of activated nociceptive ion channels depending on the type of stimuli (Dhaka et al., 2006; Patapoutian et al., 2009). Recent RNA-seq analysis highlight the fact that chemotherapeutic agents induce dramatic transcriptomic changes within the cell bodies of DRG neurons which might subsequently change the neuronal excitability that persists in pain (Starobova and Vetter, 2017). For instance, paclitaxel treatment induced up-regulation of G protein-coupled receptors and ion channels in the rat lumbar 4 and 5 (L4/5) DRGs directly supplying the sciatic nerves (Sun et al., 2023), both of which are associated with the development of neuropathic pain (Geppetti et al., 2015). In another study, up-regulation of ion channels including voltage-gated sodium channels, voltage-gated potassium channels (K_v_) and TRPs were observed in both DRGs and spinal dorsal horns of paclitaxel-treated rats (Kim et al., 2020). A recent study suggestsed that paclitaxel induced sustained activation of mammalian target of rapamycin (mTOR) and downstream MNK-eIF4E signaling pathway to substantially modify the translation efficiency of various ion channels and GPCRs in DRG neurons. Interestingly, blockade of MNK-eIF4E signaling pathways using a potent MNK inhibitor eFT508 reversed the mechanical and thermal hypersensitivity in paclitaxel-treated mice. Increased neuronal excitability was observed in paclitaxel-treated DRG neurons, a condition that could be completely inhibited by pharmaceutical blockade of MNK-eIF4E signaling using eFT508 (Megat et al., 2019). Similarly, oxaliplatin treatment significantly altered the expression of voltage-gated ion channels and genes involved in synaptic transmission in rat DRGs (Housley et al., 2020).

Functional characterization reveals the pathogenic role of nociceptive ion channel during the development of CIPN (Figure 2C). Paclitaxel induces up-regulation of Na_v_1.7 in small- and medium-sized sensory DRG neurons, as well as in sensory fiber terminals in the dorsal horns. The increased expression of Na_v_1.7 led to an increase in ectopic spontaneous neuronal activity as detected by whole-cell patch clamp in DRG neurons of paclitaxel-treated rats (Li et al., 2018). Blockade of Na_v_1.7 using a selective Na_v_1.7 blocker ProTx II markedly inhibited the spontaneously evoked action potentials in DRG neurons and alleviated paclitaxel-induced hyperalgesia in rats (Li et al., 2018). A subsequent study revealed that paclitaxel increased the number of Na_v_1.7 channels at the nerve endings by enhancing the anterograde transport of Na_v_1.7 vesicles to distal axons (Akin et al., 2021). Similarly, vincristine treatment up-regulated the expression of Na_v_1.7/Na_v_1.8 channels in rat DRG neurons (Wang et al., 2021) and spinal cord (Fu and Zhu, 2021). Pharmaceutical blockade of Na_v_1.7 using a selective blocker PF-05089771 or gastrodin (an active bioactive component of traditional Chinese medicine, Gastrodia, which is widely used for analgesic) as well as gene silencing of Na_v_1.7, greatly reduced the vincristine-induced hyperexcitability in cultured DRG neurons and reversed vincristine-induced mechanical allodynia in rats (Fu and Zhu, 2021; Wang et al., 2021). Paclitaxel treatment promoted the anterograde transport of Na_v_1.8 channels toward the distal axons, which resulted in an increased expression of Na_v_1.8 channels at the surface of the distal axons from the cultured DRG neurons (Baker et al., 2023). Oxaliplatin induced up-regulation of gene and protein expression of voltage-gated Na_v_1.6 in rat DRGs (Li et al., 2019b). While oxaliplatin induced repetitive action potential discharges in myelinated axons when electrically stimulated at 20°C, gene ablation of Na_v_1.6 did not evoke such action potential burst upon electric stimulation at the same temperature after oxaliplatin treatment, demonstrating that the induction of cold allodynia by oxaliplatin required Na_v_1.6 (Sittl et al., 2012). Another study further confirmed that Na_v_1.6 was the key sodium ion channel responsible for oxaliplatin-induced cold allodynia, as only the selective Na_v_1.6 antagonist GIIIA showed complete reversal of oxaliplatin-induced cold allodynia. Mice lacking Na_v_1.3, Na_v_1.8, or Na_v_1.9, TIIA (antagonists against Na_v_1.1, Na_v_1.2, and Na_v_1.4), and antagonists against different TRPs showed no protective effects on oxaliplatin-induced cold allodynia (Deuis et al., 2013). Similarly, blockade of Na_v_1.6 using antagonist GIIIA alleviated cisplatin-induced mechanical allodynia in rats, confirming the pathogenic role of Na_v_1.6 in cisplatin-induced mechanical allodynia (Deuis et al., 2014).

Potassium channels are the most diverse class of ion channels in neurons. In human, a total of 78 genes are encoded for potassium channels (Gonzalez et al., 2012), and they can be divided into four functionally distinct subgroups—voltage-gated potassium channels (K_v_), two-pore potassium channels (K_2P_), calcium-activated potassium channels (K_Ca_), and inward-rectifying potassium channels (K_Ir_) (Ocana et al., 2004; Tsantoulas and McMahon, 2014). Potassium channels function to inhibit neuronal excitability and counteract the initiation of action potential, and thus activation of potassium channels is thought to suppress the spontaneous activity in the DRG neurons during chronic pain (Tsantoulas and McMahon, 2014). These observations are in line with earlier studies reporting a marked reduction in the protein expression of various subunits of K_v_ in injured DRG neurons after spinal nerve ligation injury in rats (Rasband et al., 2001), indicating the involvement of potassium channels in the development of neuropathic pain after injury. Subsequent functional characterization of potassium channels reveals that mice with reduced expression of K_v_1.1 or K_v_1.2 developed neuropathic pain symptoms, including mechanical and cold allodynia (Clark and Tempel, 1998; Zhao et al., 2013). In another study, K_2P_1.1 was highly expressed in small-, medium- and large-sized DRGs (Mao et al., 2017). Similarly, K_2P_ TREK1, TREK2, and TRAAK were found to be abundantly expressed in small-sized DRGs (Alloui et al., 2006; Noel et al., 2009; Acosta et al., 2014). Since K_2P_ play crucial roles in maintaining the resting membrane potential of neurons, the down-regulation of these K_2P_ in pathogenic conditions has been shown to contribute to the development of mechanical, thermal and cold hypersensitivity (Alloui et al., 2006; Noel et al., 2009; Acosta et al., 2014; Mao et al., 2017). Interestingly, DRGs from oxaliplatin-treated mice displayed a significant down-regulation of mRNA expression of K_v_1.1 (but not K_v_1.2), together with a down-regulation of K_2P_ TREK1 and TRAAK. Co-ablation of TREK1 and TRAAK in mice recapitulated the oxaliplatin-induced mechanical and cold allodynia (Descoeur et al., 2011), highlighting the importance of these two K_2P_ in developing oxaliplatin-induced peripheral neuropathy symptoms. Similarly, paclitaxel induced down-regulation of several potassium channels, including K_Ir_1.1, K_Ir_3.4, and K_2P_1.1 (Zhang and Dougherty, 2014); however, how the changes in potassium channel expression influence the development of paclitaxel-induced peripheral neuropathy still remains elusive. KCNQ2, KCNQ3, and KCNQ5 are the major KCNQ potassium channels expressed in DRG neurons. Paclitaxel-induced hyperexcitability of DRG neurons is linked to the inhibition of KCNQ2 and systematic administration of a selective blocker of KCNQ2 (XE-991), induced mechanical allodynia and loss of IENFs (Wu et al., 2022). On the contrary, administration of a Food and Drug Administration (FDA)-approved K_v_7 opener retigabine completely prevented the rats from developing mechanical allodynia and axon degeneration after paclitaxel treatment (Li et al., 2019a). Retigabine also exhibited similar neuroprotective effects in preventing axonal loss induced by cisplatin (Nodera et al., 2011).

Calcium is one of the most common intracellular second messengers activates a specific set of calcium-dependent enzymes (Hook and Means, 2001). Calcium released from internal stores such as mitochondria and endoplasmic reticulum, and influx of extracellular calcium involve in many neurological functions, including neurotransmitter release, activation of transcription and muscle contraction (Zamponi, 2016). Voltage-gated calcium channel (Ca_v_) is a family of multisubunit transmembrane proteins that control calcium influx in response to membrane depolarization. A total of nine subtypes of Ca_v_ are expressed in the mammalian nervous system (Catterall et al., 2005). Several drugs have been identified to target Ca_v_ for treating neuropathic pain and exhibit high efficacy in pre-clinical chronic pain study (Patel et al., 2018). For instance, gabapentin and pregabalin which are known to inhibit calcium influx via the calcium channel α2δ1 subunit, effectively reduce the hyperalgesia for mechanical and thermal stimuli in various type of neuropathic pain (McGivern, 2006). Interestingly, elevated expression of α2δ1 was detected in rat DRG neurons and spinal cord after paclitaxel or oxaliplatin treatment (Kawakami et al., 2012; Yamamoto et al., 2016), suggesting that α2δ1 play a pivotal role in mediating neuropathic pain in CIPN conditions. In fact, gabapentin is shown to alleviate neuropathic pain symptoms induced by paclitaxel and oxaliplatin (Ohsawa et al., 2014; Kato et al., 2020). Clinical trials are undergoing to test the efficacy of gabapentin for pain relief in paclitaxel-induced CIPN patients. Preliminary results indicate a significant improvement in neurological outcomes (improved NCV and reversal of painful symptoms) in a small group of gabapentin-treated CIPN patients (Aghili et al., 2019), despite the fact that the neuroprotective effect remains to be determined by using a larger cohort of patients (Pandey et al., 2023).

Considerable efforts have been dedicated to unraveling the role of cation (sodium, potassium and calcium) channels in the pathogenesis of CIPN (Price et al., 2009). However, there has been a notable lack of emphasis on anion (chloride) channels, including calcium-activated chloride channels (CaCCs), voltage-gated chloride channels (Cl_v_), ligand-gated chloride channels, and volume-regulated chloride channels, on their contributions to the development of CIPN (Verkman and Galietta, 2009). In most neurons, the homeostasis of intracellular chloride ions is tightly orchestrated by the sodium-potassium-chloride co-transporter NKCC1 and the potassium/chloride co-transporter KCC2 (Payne et al., 2003; Kaila et al., 2014). NKCC1 transports chloride ions into the cells, while KCC2 extrudes the chloride ions out of the cells (Price et al., 2009). NKCC1 is abundantly expressed in the cell bodies of DRG neurons, whereas the expression of KCC2 is barely detected in these sensory neurons (Wilke et al., 2020). In contrast, both NKCC1 and KCC2 are widely expressed in the dorsal horn of the spinal cord (Payne et al., 2003; Javdani et al., 2020). A growing body of evidence suggests that dysregulation of chloride homeostasis triggers disinhibition in the spinal cord, ultimately leading to increased neuronal excitability and thereby the manifestation of hyperalgesia and allodynia (Coull et al., 2003; Price et al., 2009). Notably, paclitaxel treatment markedly increased the protein expression of NKCC1 in the dorsal spinal cord (Chen S.R. et al., 2014) and induced a significant down-regulation of KCC2 mRNA expression in the dorsal horn neurons (Yeo et al., 2022). Elevating the expression of KCC2 in the dorsal spinal cord or enhancing KCC2 activity through phosphorylation in the dorsal horn neurons restored chloride homeostasis and alleviated pain hypersensitivity in rats (Ford et al., 2015; Li et al., 2016; Ouyang et al., 2019), suggesting that KCC2 might be a potential therapeutic target for CIPN. However, it remains unclear whether other CIPN-causing agents also induce changes in NKCC1 and KCC2 expression within the dorsal spinal cord. Substantial efforts are required to uncover the role of other chloride channels in the pathogenesis of CIPN.

TRP channels represent the largest group of noxious stimulus detectors and emerging targets for novel analgesic development (Patapoutian et al., 2009). In the past decades, multiple TRP channels have been identified as novel therapeutic targets for CIPN (Naziroglu and Braidy, 2017). TRPA1 channel is the central chemical-sensing receptors (Tai et al., 2008), and found to be co-expressed with TRPV1 in a subset of DRG and trigeminal neurons for sensing noxious stimuli (Story et al., 2003). Both TRPA1 and TRPV1 are crucial receptors for thermosensation and serve as the primary transducers of thermal stimuli (Caterina et al., 1997; Story et al., 2003). TRPM8, on the other hand, is expressed in a separate subset of DRG neurons that are responsible for the detection of cold stimuli (Peier et al., 2002). After cisplatin or oxaliplatin treatment, the mRNA expression of both TRPA1 and TRPV1 was markedly increased in the rat DRG neurons (Ta et al., 2010; Descoeur et al., 2011; Khasabova et al., 2012; Yamamoto et al., 2015). Genetic ablation of TRPV1 substantially decreased cisplatin-induced thermal hypersensitivity (Ta et al., 2010). Similarly, intrathecal injections of antisense oligodeoxynucleotides specifically targeting TRPA1 alleviates oxaliplatin-induced cold hypersensitivity (Yamamoto et al., 2015). Microtubule-targeting chemotherapeutic agents, paclitaxel and vincristine, up-regulated the protein expression of TRPV1 in rat DRGs (Hara et al., 2013; Chiba et al., 2017). A TRPV1 blocker capsazepine, alleviated the mechanical and cold allodynia induced by paclitaxel and vincristine (Hara et al., 2013; Chiba et al., 2017). Interestingly, a TRPA1 blocker HC-030031 reversed mechanical and cold allodynia induced by paclitaxel and bortezomib; however, there is no direct evidence demonstrating an up-regulation of TRPA1 after both chemotherapy drug treatments (Materazzi et al., 2012; Trevisan et al., 2013). TRPM8 is another example of the lack of direct correlation between expression level and CIPN symptoms, despite the fact that TRPM8 expression was increased in DRGs after cisplatin and oxaliplatin treatment, but the roles of TRPM8 in CIPN development remain largely unknown (Gauchan et al., 2009; Ta et al., 2010; Kawashiri et al., 2012).

To this end, although the altered expression of ion channels and neuronal excitability in DRG neurons might be a common CIPN pathophysiology, a more systematic approach is necessary for the identification of key upstream regulators causing such transcriptomic changes that might serve as better therapeutic targets for CIPN.

### 3.4 Neuroinflammation

Another major CIPN-related pathophysiological mechanism involves the activation of immune system, resulting in neuroinflammation and degeneration of primary afferent sensory fibers (Starobova and Vetter, 2017; Figure 2D). After paclitaxel or vincristine treatment, invasion of activated tissue-resident macrophages (i.e., Langerhans cells) into the epidermis of glabrous hindpaw skin was found (Siau et al., 2006). The increase of Langerhans cells in the hindpaw skin is thought to cause the loss of IENFs due to the local production of neurotoxic pro-inflammatory cytokines (Wang et al., 2012). Administration of anti-inflammatory agent such as minocycline (Garrido-Mesa et al., 2013), protected the IENFs from paclitaxel-induced axon degeneration and mechanical hyperalgesia (Boyette-Davis et al., 2011). Similarly, oxaliplatin triggered the activation of cutaneous mast cells (another type of tissue-resident immune cells) via the activation of proteinase-activated receptor 2 (PAR2), and induced IENF degeneration and mechanical allodynia. Oxaliplatin-induced peripheral neuropathy can be prevented by depleting mast cells or inhibiting PAR2 by antagonist FSLLRY-NH_2_ (Sakamoto et al., 2016).

Apart from local inflammation, activation of glial cells (satellite glial cells, Schwann cells and immune cells) in the DRGs are also involved in the augmented inflammatory responses and enhanced neuronal excitability resulting in the development of pain hypersensitivity after chemotherapy treatment (Fumagalli et al., 2020). Following paclitaxel or oxaliplatin treatment, an elevated immunoreactivity to glial fibrillary acidic protein (GFAP), a marker for satellite and unmyelinated Schwann cells, was detected in rat DRGs (Peters et al., 2007; Warwick and Hanani, 2013). In parallel, a massive number of infiltrating macrophages was observed in DRGs, sciatic nerves and dorsal horns of the spinal cord (Peters et al., 2007; Zhang et al., 2016; Illias et al., 2022). The expression of pro-inflammatory cytokines (IL-1β and TNF-α) was markedly increased in the DRGs (Ledeboer et al., 2007; Zhang et al., 2016) and dorsal horns of the spinal cord (Janes et al., 2015). Paclitaxel or oxaliplatin treatment also promoted the production of chemokines (CCL2, CCL3, CCL4, and CCL11) in both DRGs and spinal cord (Makker et al., 2017). Blockade of pro-inflammatory cytokine receptor (IL-1ra) as well as intrathecal injection of anti-inflammatory cytokine (IL-10) while systematic ablation of infiltrating macrophages using liposomal clodronate that greatly reduced the production of pro-inflammatory cytokines, all these protected the loss of IENFs and reversed the paclitaxel-induced mechanical allodynia (Ledeboer et al., 2007; Zhang et al., 2016).

### 3.5 Central mechanisms of CIPN

Since the concept of central sensitization was first described in 1983 (Woolf, 1983), an increasing body of evidence has indicated that the development of chronic pain, including CIPN, is predominantly attributed to central rather than peripheral mechanisms (Woolf and Salter, 2000; Latremoliere and Woolf, 2009; Nijs et al., 2021). In contrast to peripheral sensitization where reduction in pain threshold and amplification in nociceptor responsiveness occurs in the peripheral axonal terminals (Hucho and Levine, 2007), central sensitization refers to heightened nociceptor responsiveness in the CNS and spinal dorsal horn to afferent inputs, leading to hypersensitivity to suprathreshold stimuli, increased responsiveness to innocuous stimuli, and enlarged receptive fields (Woolf and Salter, 2000). The intense, repetitive, and sustained noxious stimuli activate C-fiber nociceptors and cause a sustained release of fast neurotransmitter glutamate, which then binds to NMDA receptors on postsynaptic neurons in the dorsal horn of the spinal cord, inducing central sensitization (Woolf and Thompson, 1991). Inhibition of NMDA receptors using non-competitive antagonist MK801, competitive antagonist D-CPP, or conditional deletion of the NR1 subunit of NMDA receptors in the dorsal horn of the spinal cord effectively reverses the hyperexcitability in nociceptive neurons, thereby abolishing activity-dependent central sensitization (Woolf and Thompson, 1991; Ma and Woolf, 1995; South et al., 2003). Collectively, these findings underscore the significance of NMDA receptors and offer clinical insights into potential treatments for chronic pain caused by central sensitization.

While considerable efforts have been directed toward understanding the peripheral mechanisms of CIPN, emerging evidence indicates that CIPN-causing agents also induce changes in CNS neurons, leading to the development of central sensitization (Figure 2E). Studies have proposed that chemotherapeutic agents including cisplatin, oxaliplatin and paclitaxel cause dysfunction of the blood–brain barrier (Wardill et al., 2016). After a single intraperitoneal administration of oxaliplatin to rats, a trace amount of oxaliplatin (approximately 6.6 nM) was detected in the cerebrospinal fluid (CSF) of the rats. It resulted in increased activity in the dorsal horn activity and the development of mechanical allodynia in those rats, when an equivalent concentration of oxaliplatin was directly applied to the spinal dorsal surface. The increased neuronal excitability in the dorsal horn of the spinal cord was at least partly attributed to the elevated level of chemokines CXCL3, as administration of CXCL3-neutralizing antibodies abolished the hyperexcitability in those neurons (Huang et al., 2016). Notably, CIPN-causing agents induced widespread activation of microglia and astrocytes, leading to enhanced inflammatory responses in the dorsal spinal cord (Janes et al., 2015; Makker et al., 2017; Di Cesare Mannelli et al., 2022). Inflammatory mediators, including TNF-α, IL-1β, IL-6 and neuropeptide substance P, have been implicated in the neuronal hyperexcitability of the spinal dorsal horn and the development of central sensitization (Wiesenfeld-Hallin and Xu, 1993; Detloff et al., 2008; Costigan et al., 2009; Konig et al., 2016). Blockade of the microglial P2X7 receptors or administration of minocycline, which has potent anti-inflammatory properties, has been shown to attenuate the production of these inflammatory mediators and alleviated chronic pain symptoms caused by neuronal hyperexcitability in the spinal dorsal horn (Chu et al., 2010; Konig et al., 2016).

The spinal dorsal horn neurons project their axons to the thalamus. The sensory outputs from the thalamus are then conveyed into the amygdala and the anterior cingulate cortex (ACC) for the processing of pain perception (Zhuo, 2006; Bliss et al., 2016). In response to noxious mechanical stimuli, the glutamatergic pyramidal neurons in the ACC become excited, which results in a release of neurotransmitter GABA in adjacent inhibitory interneurons through a feedback loop mechanism (Hutchison et al., 1999; Iwata et al., 2005). Chronic pain triggers a sustained increase in neuronal excitability and synaptic potentiation in the ACC, accompanied by elevated activity of the glutamate receptor GluR1 (Wu et al., 2005; Xu et al., 2008) (Figure 2E). Recent studies highlight that CIPN-causing agents induce changes in the functional connectivity within the ACC and subcortical periaqueductal gray, as observed in animal models and human studies (Boland et al., 2014; Ferris et al., 2019). Paclitaxel, for example, induced up-regulation of activity-dependent immediate early gene c-Fos in the ACC (Cao et al., 2022), which is a common phenomenon in other chronic pain conditions (Li et al., 2010; Chen T. et al., 2014). Paclitaxel also altered the gene expression of GABA transporter-1 in the ACC (Masocha, 2015), resulting in lower availability of GABA in the post-synaptic terminals and increased neuronal excitability (Nashawi et al., 2016). Interestingly, chemogenetic or optogenetic inhibition of the glutamatergic neurons in the ACC, or exogenous administration of GABA, has been found to abolish paclitaxel-induced neuronal hyperexcitability and mechanical allodynia (Nashawi et al., 2016; Cao et al., 2022). To this end, modulating neuronal plasticity in the ACC holds immense promise for alleviating neuropathic pain, and further investigation is therefore needed to explore novel therapeutic agents, such as PKMζ inhibitors (Li et al., 2010), in alleviating ACC hyperexcitability and CIPN symptoms.

### 3.6 Influence of gut microbiota in the pathogenesis of CIPN

The complex crosstalk between gut microbiota, CNS and the enteric nervous system constitutes the gut-brain axis that influences cognition, emotion, memory and learning, and motor coordination (Carabotti et al., 2015; Morais et al., 2021). It is well documented that deleterious changes to the composition of gut microbiota possess a significant impact on the CNS, which could eventually lead to various neurological disorders, such as autism (Mayer et al., 2014), Alzheimer’s disease (Jiang et al., 2017; Kowalski and Mulak, 2019), and Parkinson’s disease (Scheperjans et al., 2015; Unger et al., 2016). In recent years, there is mounting evidence suggesting the contribution of gut microbiota to the pathological condition of pain (Lin et al., 2020; Pane et al., 2022). In mice, a spared nerve injury (SNI) induced a drastic change in the microbiota composition and the abundance of *Staphylococcus* sp. increased significantly at the time point when severe mechanical allodynia was observed (Brandon-Mong et al., 2020). Similarly, chronic constriction injury (CCI) promotes the relative abundance of *Streptococcus*, *Lactobacillus*, *Helicobacter*, *Blautia*, *Christensenella*, and *Phascolarctobacterium* in the gut microbiota. The altered gut microbiota significantly changed the serum metabolomics profile of the CCI rats. Further in-depth network analysis revealed that the change in gut microbiota and serum metabolomics correlated well with the impaired lipid metabolism, molecular transport and augmented inflammatory responses in CCI rats (Chen et al., 2021). Interestingly, antibiotic treatment aiming to remove gut microbiota prevented the development of CCI-induced mechanical and thermal hyperalgesia. After antibiotic treatment, the number of interferon-gamma (IFN-γ) type 1 T helper (Th1) cells (responsible for the production of pro-inflammatory IFN-γ and TNF-α, and activated macrophages) was markedly reduced and the number of regulatory T cells (suppress and limit chronic inflammation) was increased in CCI mice. The antibiotic-induced neuroprotective effects were abolished if regulatory T cells were depleted in the CCI mice, suggesting that gut microbiota influences the inflammatory responses and regulates pain hypersensitivity (Ding et al., 2021).

It is well-documented that 129S6/SvEvTac mice exhibit notably less sensitivity to nociceptive pain compared to the widely used C57BL/6 mice (Lariviere et al., 2001; Leo et al., 2008). Spinal nerve ligation (SNL) injury induced pain hypersensitivity in C57BL/6 mice partly due to the profound up-regulation of dynorphin (a protein known to induce chronic pain) (Podvin et al., 2016) in the spinal cord. Interestingly, 129S6/SvEvTac mice fail to induce up-regulation of dynorphin in the spinal cord after SNL and thus do not display pain hypersensitivity in this model (Gardell et al., 2004). A recent study suggests that paclitaxel-induced peripheral neuropathy symptoms largely depend on the composition of the gut microbiota (Ramakrishna et al., 2019). Paclitaxel induces mechanical, thermal, and cold hyperalgesia in C57BL/6 mice, while 129S6/SvEvTac mice are resistant to the development of CIPN symptoms after paclitaxel treatment. Interestingly, antibiotic treatment that depleted gut microbiota in C57BL/6 mice and protected the mice from developing CIPN symptoms after paclitaxel treatment. Moreover, 129S6/SvEvTac mice received fecal microbiota transplantation from C57BL/6 mice developed mechanical, thermal and cold hyperalgesia after paclitaxel treatment (Ramakrishna et al., 2019). Particularly, paclitaxel treatment markedly elevated the relative abundance of *Alistipes onderdonkii* and reduced the relative abundance of *Akkermansia muciniphila* (Ramakrishna et al., 2019). The elevated abundance of bacterial phylum *Alistipes* is shown to be associated with fibromyalgia, a chronic medical condition that caused widespread musculoskeletal pain (Clos-Garcia et al., 2019). Restoring the abundance of *Akkermansia muciniphila* attenuated neuroinflammation mouse model of Parkinson’s Disease and ameliorated inflammation in chronic colitis model (Qiao et al., 2024; Yilmaz et al., 2024), suggesting that the decreased abundance of *Akkermansia muciniphila* might correlate well with the augmented neuroinflammation to mediate pain hypersensitivity after paclitaxel treatment. Similarly, oxaliplatin induces persistent mechanical allodynia in mice, a condition that can be completely reversed by eliminating the gut microbiota after antibiotic treatment, suggesting that the change in gut microbiota (dysbiosis) contributed to the development of CIPN induced by oxaliplatin. The 16S rRNA gene sequencing and phylum analysis revealed an increased relative abundance of *Verrucomicrobia* and drastic change in the ratio of *Bacteroidetes* and *Firmicutes* (Shen et al., 2017). Accumulating evidence suggested that the imbalance of *Bacteroidetes* and *Firmicutes* ratio contributed to the development of chronic pain (Garvey, 2023). In another study, elevated abundance of *Verrucomicrobia* was linked to chronic abdominal pain (Kang et al., 2019). Interestingly, antibiotic treatment not only attenuated the expression level of pro-inflammatory cytokines IL-6 and TNF-α, but also reduced the infiltration of macrophages into the DRGs of oxaliplatin-treated mice. Administration of lipopolysaccharide (LPS)-derived from gut microbiota completely abolished the antibiotic-induced neuroprotective effects in oxaliplatin-treated mice, possibly via the ligand binding of LPS to its own receptor toll-like receptor 4 (TLR4) (Shen et al., 2017). In fact, therapeutic interventions aim at improving the gut microbiota have already demonstrated promising results in alleviating neuropathic pain (Guo et al., 2019). For instance, a novel probiotic formulation called SLAB51 protected the mice from developing mechanical and cold hypersensitivity after paclitaxel treatment (Cuozzo et al., 2021). Therefore, further investigation is required to fully understand the causal relationship between gut microbiota composition and neuroinflammation, as well as their contributions to the pathogenesis of CIPN (Figure 2F).

## 4 Future perspective: targeting the right source for improved therapeutics

Despite tremendous research effort has been devoted to understanding the mechanisms underlying CIPN pathogenesis, there are still no effective drugs that can prevent the development of CIPN (Wolf et al., 2008; Staff et al., 2017). In the past decades, breakthroughs in targeted drug therapy led to a rapid development of new generation of chemotherapeutic agents with increased efficacy and reduced side effects (Pucci et al., 2019; Debela et al., 2021). However, the traditional CIPN-causing chemotherapeutic drugs remain as major treatment options if the new drugs fail to show sustain efficacy or the patients cannot afford expensive new medicines. Dose reduction and discontinuation of these life-saving treatments are the only options for the cancer patients who develop CIPN. Development of novel CIPN-preventing agents remains a highly challenging task and an unmet medical need for cancer patients. By minimizing the off-target neurotoxic effects that overlap with cancer cytotoxic mechanisms, it might also reduce the drug efficacy. Therefore, strategies for preventing CIPN must be neuronal-specific with minimal interference with the anti-tumor effects of the cancer drugs in the first place.

Targeting the increased neuronal excitability in sensory neurons using a wide variety of anticonvulsants (e.g., gabapentin, lamotrigine, or pregabalin) is originally considered as a promising approach for CIPN (Starobova and Vetter, 2017). Unfortunately, many of these anticonvulsants have no known or limited beneficial effects on pain management in clinical trials (Rao et al., 2007, 2008; Saif et al., 2010). However, the use of anticonvulsants for pain management might need extra caution due to the abundant expression of sodium and potassium channels in the nervous systems. Anticonvulsant drugs have been found to exhibit a range of side effects, including dizziness, nausea, vomiting, blurred vision, mood swing, reduced sexual performance, somnolence and even loss of cognitive functions (Patapoutian et al., 2009). To this end, further investigation is required to identify potent neuroprotective and pain relief agents without compromising the effectiveness of systemic antineoplastic drug regimens.

Bulk tissue RNA sequencing of DRG reveals transcriptomic changes in a mixture of different neuronal subtypes and non-neuronal cells. In contrast, recent advance in single-cell RNA sequencing (scRNA-seq) allows the characterization of transcriptomic changes across different functionally distinct cell types down to single-cell resolution in DRGs. Recently, scRNA-seq revealed heterogenous, subtype-specific transcriptomic changes in DRGs (Hu et al., 2016; Zhang C. et al., 2022) and retinal ganglion cells (Jacobi et al., 2022; Li et al., 2022) after axotomy. The distinct gene expression changes in different neuronal subtypes might account for the differences in the intrinsic growth capacity of different neuronal subtypes.

A recent scRNA-seq study suggests that paclitaxel-induced transcriptional changes are different from the gene expression pattern of injury-induced neuropathic pain, despite the involvement of macrophages infiltration in the development of CIPN in both peripheral nerve injury models (Renthal et al., 2020). A better understanding of the transcriptomic changes in neuronal and non-neuronal cells induced by different CIPN-causing chemotherapy agents not only could characterize a common pathophysiological mechanism underlying CIPN, but also provides a platform to identify novel therapeutics targets and preventative strategies for CIPN. In fact, we and others successfully utilized the transcriptomic perturbations as gene signatures and successfully identifies bioactive small molecules which can promote axon regeneration and function recovery after CNS injury (Chandran et al., 2016; Au et al., 2022b). With the availability of substantial transcriptional data generated from different CIPN models in recent years, we believe that by using a similar systems biology approach would facilitate the identification a common core transcriptional program and *in silico* screening in CIPN drug discovery.

## Author contributions

XC: Writing – original draft. YG: Writing – original draft. NPBA: Funding acquisition, Project administration, Supervision, Writing – review & editing. CHEM: Funding acquisition, Project administration, Supervision, Writing – review & editing.
